# Choroid plexus enlargement is negatively associated with cognitive performance in a DTI-ALPS–dependent manner

**DOI:** 10.3389/fnagi.2026.1883244

**Published:** 2026-07-27

**Authors:** Nicole I. Y. Z. Tan, Thomas Welton, Yi Jayne Tan, Seyed Ehsan Saffari, Elenor Morgenroth, Sarah J. Y. Foo, Zi Rou Kwok, Yu Chin Lim, Hui Jin Chiew, Kok Pin Ng, Simon K. S. Ting, Sumeet Kumar, Nicole Keong, Adeline S. L. Ng

**Affiliations:** 1 Department of Neurology, National Neuroscience Institute, Singapore, Singapore; 2 Neuroscience Academic Clinical Programme, Duke NUS Medical School, Singapore, Singapore; 3 Lee Kong Chian School of Medicine, Nanyang Technological University, Singapore, Singapore; 4 Department of Neuroradiology, National Neuroscience Institute, Singapore, Singapore; 5 Department of Neurosurgery, National Neuroscience Institute, Singapore General Hospital, Singapore, Singapore

**Keywords:** Alzheimer’s disease biomarkers, cerebrospinal fluid dynamics, choroid plexus, cognitive impairment, diffusion MRI, DTI-ALPS, glymphatic system, plasma pTau217

## Abstract

**Background:**

Disturbances in brain fluid homeostasis are increasingly implicated in neurodegeneration. Imaging measures of structural alterations of the choroid plexus (CP) and impaired glymphatic transport have each been associated with cognitive decline, yet their potential interaction in humans remains poorly understood.

**Methods:**

We investigated the relationship between CP volume, glymphatic diffusion, and cognitive performance in 100 memory clinic patients. Diffusion tensor imaging analysis along the perivascular space (DTI-ALPS) was used as an imaging proxy of glymphatic diffusion, and CP volume and WMH volume were derived from structural MRI using FastSurfer segmentation. Multivariable linear regression models examined associations between CP volume, ALPS index, and global cognitive performance measured by the Montreal Cognitive Assessment (MoCA) and Mini-Mental State Examination (MMSE). Models were adjusted for age, sex, education, APOE ε4 status, WMH burden and plasma phosphorylated tau (pTau217). Interaction terms tested whether CP structure and glymphatic diffusion jointly influenced cognition.

**Results:**

Larger CP volume was associated with lower ALPS index after adjustment for demographic and molecular covariates (*β* = −282.19, *p* = 0.015). CP volume and ALPS index were not independently associated with MoCA scores; however, a significant interaction between CP volume and ALPS index was observed (*β* = −13,299.09, *p* = 0.036). The association between CP volume and cognitive performance depended on DTI-ALPS, such that larger CP volumes were associated with poorer MoCA scores at higher ALPS values, whereas CP volume showed little association with cognition at lower ALPS values. This interaction improved model fit compared with main-effects models (*R^2^* = 0.31). The findings remained significant after adjusting for CSF volume and were replicated using MMSE as the outcome. Plasma pTau217 levels were strongly associated with worse cognition but did not significantly modify the CP-ALPS interaction.

**Conclusion:**

CP enlargement is associated with glymphatic diffusion, and its relationship with cognitive performance varies across DTI-ALPS index values. These findings suggest that interactions between CSF regulatory systems may correlate with cognitive performance in a state-dependent manner. Notably, higher ALPS values in individuals with enlarged CP may reflect compensatory or altered perivascular fluid dynamics rather than preserved glymphatic function, highlighting the complexity of interpreting diffusion-based markers of brain clearance.

## 1 Introduction

Neurodegenerative disorders such as Alzheimer’s disease (AD) are increasingly understood to involve disturbances in brain homeostatic systems that regulate cerebrospinal fluid (CSF) production, circulation, and clearance (Scheltens et al., 2021; Tarasoff-Conway et al., 2015; Uchida, 2022). While the accumulation of amyloid-*β* and tau proteins remains a defining feature of AD pathology, growing evidence suggests that impaired brain fluid dynamics may contribute to the development and progression of cognitive decline (Harrison et al., 2020; Peng et al., 2016). Inefficient clearance of metabolic waste can lead to the accumulation of neurotoxic proteins in brain tissue (Jessen et al., 2015), potentially accelerating neuronal dysfunction.

Two systems play central roles in maintaining this fluid balance. The choroid plexus (CP) is the primary site of CSF production and forms the blood-CSF barrier, regulating molecular exchange between the circulation and the central nervous system (Damkier et al., 2013). In parallel, the glymphatic system supports the clearance of interstitial solutes through perivascular pathways, enabling the removal of metabolic by-products from the brain (Bacyinski et al., 2017). Experimental and clinical studies suggest that dysfunction in either system may contribute to neurodegenerative processes (Preziosa et al., 2025; Xu et al., 2024; Zahran et al., 2026), but their potential interaction remains poorly understood.

Recent neuroimaging developments allow these processes to be investigated *in vivo*. The diffusion tensor imaging analysis along the perivascular space (DTI-ALPS) index has been proposed as a non-invasive surrogate of glymphatic transport efficiency, with lower ALPS values interpreted as reflecting reduced diffusion along perivascular pathways (Taoka et al., 2024). Several studies have reported lower ALPS indices in neurodegenerative disorders and associations with cognitive impairment (Hsu et al., 2023; Huang et al., 2024; Satpathi et al., 2025). At the same time, structural alterations of the CP have emerged as a potential imaging marker of brain pathology. Enlargement of the CP has been observed in individuals across the AD continuum and has been linked to neuroinflammatory processes and altered CSF dynamics (Umemura et al., 2024).

Despite these observations, the relationship between CP structural changes and glymphatic function remains unclear (Lv et al., 2026). Most previous studies have examined these markers independently, leaving unresolved whether CP enlargement and glymphatic dysfunction represent parallel processes or components of a shared pathway affecting brain clearance mechanisms. Because the CP regulates CSF production while the glymphatic system facilitates waste removal, disruption of both systems may interact to correlate with cognitive outcomes.

Interpretation of such imaging findings is complicated by the presence of established molecular biomarkers of neurodegeneration. Plasma phosphorylated tau (pTau217) has recently emerged as a sensitive marker of AD pathological burden and shows strong associations with cognitive impairment (Fernández Arias et al., 2025; Gonzalez-Ortiz et al., 2025; Malek-Ahmadi et al., 2026). Determining whether imaging markers related to CSF dynamics provide additional information beyond molecular pathology may therefore help improve their prognostic utility.

In this study, we tested the primary hypothesis that structural alterations of the CP and functional changes in glymphatic diffusion jointly correlate with cognitive performance. Specifically, we hypothesized that larger CP volume would be associated with reduced glymphatic diffusion and that the combination of CP enlargement and impaired glymphatic diffusion would be associated with poorer cognitive outcomes. The aim of this study was therefore to determine whether these two imaging markers interact to correlate with global cognitive performance in a memory clinic cohort, while accounting for established AD pathology measured using plasma pTau217. We supplemented this with confirmatory analysis, testing the sensitivity of the main model results to potentially confounding factors.

## 2 Materials and methods

### 2.1 Participants

Participants were recruited from memory clinics at the National Neuroscience Institute, Singapore between 2023 and 2025. Adults aged 21 years and above were included in the study. Exclusion criteria were MRI contraindication, and the presence of other neurological, neuropsychiatric or neurosurgical conditions. All participants underwent MRI, neurological evaluation, cognitive testing and blood draw in a single study visit. Demographic variables including age, sex, and education were recorded for all participants.

### 2.2 Ethical approval

All participants provided written informed consent to study protocols approved by the Singapore Health Services Centralized Institutional Review Board (CIRB; reference number 2017-2602 and 2023-2569). The research was conducted in accordance with the Declaration of Helsinki and the Belmont Report.

### 2.3 Cognitive assessment

Cognitive assessment included global cognitive screening using the Mini-Mental State Examination (MMSE) and Montreal Cognitive Assessment (MoCA).

### 2.4 MRI acquisition

MRI data were acquired on a 3-Tesla Philips Ingenia scanner. A clinical diffusion tensor imaging sequence was obtained using the following parameters: slice thickness 2 mm, voxel size 2 × 2 mm, matrix 110 × 110, echo time (TE) = 86 ms, repetition time (TR) = 4.53 s, flip angle = 90°, with 8 b = 0 images and 32 diffusion-weighted directions at b = 1,000 s/mm^2^, acceleration factor = 2, and total scan time = 158 s.

High-resolution sagittal T1-weighted structural images were acquired using a 3D gradient-echo sequence with 1 mm isotropic resolution, slice thickness = 1 mm, TR = 7.29 ms, TE = 3.32 ms, flip angle = 8°, acceleration factor = 2, and total scan time = 386 s.

### 2.5 Diffusion MRI processing and ALPS index calculation

Diffusion MRI data were preprocessed using MRtrix3 (version 3.0.8) (Tournier et al., 2019). Noise was removed using Marchenko-Pastur principal component analysis denoising (dwidenoise), followed by Gibbs ringing correction (mrdegibbs). Motion and eddy current distortions were corrected using dwifslpreproc. A brain mask was generated using dwi2mask, and used to remove non-brain tissues from the images. Diffusion tensors were fitted using dwi2tensor. Fractional anisotropy (FA) maps and color FA images were derived using tensor2metric.

The DTI-ALPS index was calculated using a standardized approach (Taoka et al., 2024). Regions of interest (ROIs) were manually placed by a neuroradiologist blinded to clinical information. Moving from inferior to superior axial slices, we selected the first slice in which both projection and association bundles were visible in both left and right hemispheres, and large enough to contain the ROIs. ROIs were positioned on projection and association fiber areas adjacent to the lateral ventricles in both hemispheres on color FA maps. ROIs were placed to avoid regions of crossing fibers. We avoided partial-volume contamination effects by careful placements of ROIs to avoid boundaries and by selecting the ROI size to not exceed the width of the bundles. ROIs comprised four voxels placed in a square on an axial slice, each having a volume of 12.17 mm^3^. Diffusivity along the x-, y-, and z-axes was extracted from each region (Supplementary Figure 1). The ALPS index was then calculated as:


$$\mathrm{DTI}-\text{ALPS}=\frac{\mathrm{Dxx}.\text{proj}+\mathrm{Dxx}.\text{assoc}}{\mathrm{Dyy}.\text{proj}+\mathrm{Dzz}.\text{assoc}}$$

The mean of the ALPS index from both hemispheres was used in our analyses. Lower ALPS values correspond to lower diffusivity along perivascular spaces and have been proposed as an indirect imaging marker of glymphatic diffusion (Satpathi et al., 2025). The reliability of the ROI placement procedure was assessed in an independent set of 40 MRI scans, for which ROIs were independently placed by three raters. Inter-rater reliability was assessed using a two-way mixed-effects intraclass correlation coefficient (ICC) with single measures and an absolute agreement definition. Inter-rater reliability was good for single-measure ALPS indices (ICC = 0.882, 95% CI [0.812–0.931]; reflecting the reliability of measurements obtained by a single rater) and excellent for average-measure indices (ICC = 0.957, 95% CI [0.928–0.976]; reflecting the reliability of the mean measurement across raters) (Koo and Li, 2016), supporting the reproducibility of the manual ROI placement method.

### 2.6 Choroid plexus and cerebrospinal fluid volume estimation

T1-weighted structural images were processed using FastSurfer (version 7.4.1) (Henschel et al., 2020), an automated deep learning-based neuroimaging pipeline. This pipeline generated volumetric segmentations including the CP, ventricular CSF, and estimated total intracranial volume (eTIV). CP volume was derived from the left and right CP labels generated by FastSurfer and were summed from both hemispheres. These labels primarily represent CP within the lateral ventricles (Supplementary Figure 2). Ventricular CSF volume was calculated as a sum of the lateral ventricles, inferior lateral ventricles, third ventricle, and fourth ventricles from both hemispheres. All segmentations were visually inspected for accuracy by a trained researcher, and scans with major segmentation errors were excluded prior to analysis. Segmentations were visually inspected in multiple planes to identify gross misclassification, under-segmentation of CP tissue, and over-segmentation into adjacent ventricular CSF or surrounding brain parenchyma. No scans required exclusion or manual correction following quality control.

### 2.7 Plasma biomarker measurement

Blood samples were collected into EDTA tubes and centrifuged at 1,800 g for 10 min within one hour of collection. Plasma was aliquoted and stored at −80 °C until analysis. Plasma pTau217 concentrations were measured using ALZpath SIMOA pTau217 Advantage PLUS kits (Quanterix, Massachusetts, United States) on the HDX Analyzer according to the manufacturer’s protocol. Inter- and intra-assay coefficients of variation were <15%.

### 2.8 APOE genotyping

Apolipoprotein E (APOE) genotype was determined in all participants as described previously (Tan et al., 2016).

### 2.9 Statistical analysis

All statistical analyses were performed using R (version 4.3.1; 2023-06-16). Statistical significance was defined as a two-sided *p* value <0.05. Four baseline patient data had to be excluded due to missing APOE ε4 carrier status and cognitive data (Supplementary Figure 3).

CP and CSF volumes were normalized by dividing by eTIV to account for interindividual differences in brain size. Plasma pTau217 values showed right-skewed distributions and were therefore log-transformed prior to analysis. Continuous predictors used in regression models were mean-centered to facilitate interpretation of interaction terms.

For descriptive purposes, participants were stratified into previously defined plasma pTau217 risk strata (low <0.38 pg./mL, indeterminate 0.38–0.66 pg./mL, high >0.66 pg./mL) based on cut-offs derived from prior work in this cohort (Tan et al., 2026). However, pTau217 was treated as a continuous variable in regression analyses.

We fitted a single main primary regression model and several confirmatory sensitivity models. The regression models examined relationships between imaging markers and cognitive outcomes. For each model, we inspected model assumptions, including the normality of residuals using Q-Q plots.

First, before testing the main interaction model, we tested the association between CP volume and glymphatic diffusion was assessed using a regression model with the ALPS index as the dependent variable. To evaluate relationships with cognition, regression models were constructed with MoCA scores as the primary outcome and MMSE scores as a secondary outcome. All models were adjusted for age, sex, education, APOE ε4 carrier status, and log-transformed plasma pTau217 levels. These covariates were selected *a priori* based on known associations with cognitive performance and AD pathology.

For our main primary hypothesis, we tested whether CP structure and glymphatic diffusion jointly correlate with cognitive performance, an interaction term (CP × ALPS) was included in regression models. Multicollinearity was assessed using variance inflation factors.

Sensitivity confirmatory analyses were conducted to examine the robustness of findings. First, normalized CSF volume was added to regression models to account for potential confounding by ventricular enlargement or global brain atrophy. Second, models were repeated separately in participants with and without clinical AD diagnoses. Third, stratified analyses were performed according to plasma pTau217 levels using previously defined thresholds (Tan et al., 2026). Fourth, we performed sensitivity analysis accounting for WMH burden. Finally, a three-way interaction model was used to evaluate whether plasma pTau217 concentration modified the relationship between CP volume, ALPS index, and cognitive performance.

Model coefficients, confidence intervals, and goodness-of-fit statistics were extracted for reporting, and figures illustrating key associations were generated using ggplot2.

## 3 Results

### 3.1 Sample characteristics

A total of 100 memory clinic patients were included in the analysis (Table 1). The cohort comprised participants across diagnostic groups, including subjective cognitive impairment (SCI; 11%), mild cognitive impairment (MCI; 70%), Alzheimer’s disease (AD; 15%), vascular dementia (VaD; 3%), and frontotemporal dementia (FTD; 1%).

**Table 1:** Demographic, clinical, cognitive and imaging characteristics of the overall memory clinic sample used in this study.

Characteristics	Overall
*N*	100
Age, mean (SD)	68.02 (7.91)
Gender, number of males (%)	50 (50.0)
Education, mean (SD)	13.27 (3.90)
APOE genotype, number of ε4 carriers (%)	29 (29.9)
Choroid plexus volume mm^3^, mean (SD)	1,715.18 (416.27)
ALPS, mean (SD)	1.44 (0.27)
CSF volume mm^3^, mean (SD)	40,282.89 (18,485.84)
White matter hyperintensity burden, mm^3^	5,386 (9,350)
MoCA, mean (SD)	24.67 (4.37)
MMSE, mean (SD)	26.32 (3.47)
pTau217 plasma concentration pg./mL, mean (SD)^a^	0.68 (0.72)

a Cut-off thresholds for pTau risk groups: low: <0.38 pg/mL; indeterminate: 0.38–0.66 pg/mL; high: >0.66 pg/mL. Association between CP volume and ALPS index.

The cohort demographics were typical of memory clinic populations, with a mean age of 68.01 (± 7.91) years, and equal proportions of males and females. Global cognitive performance ranged broadly across the cohort, with both MoCA (24.67 ± 4.37) and MMSE (26.32 ± 3.47) scores reflecting a spectrum from normal cognition to dementia. APOE ε4 genotype carrier status (29.9%) and plasma pTau217 levels (0.68 ± 0.72 pg./mL) were elevated in this sample, as expected, with a wide range of plasma pTau217 concentrations. WMH burden was mild to moderate (5,386 ± 9,350) typical for this population.

The cohort was stratified according to plasma pTau217 levels using previously derived cut-offs to indicate risk strata for AD pathological burden (low: <0.38 pg./mL; indeterminate: 0.38–0.66 pg./mL; high: >0.66 pg./mL). Using this classification, there were 51 low-risk, 16 indeterminate, and 33 high-risk participants (Supplementary Table 1). Compared to the low AD pathology risk group, participants in the high-risk group were modestly older (69.19 ± 5.91 years vs. 67.12 ± 8.71 years) and had a higher proportion of APOE ε4 carriers (53.1% vs. 16.3%). Other demographic and imaging variables showed no clear systematic differences between pTau217-based groups.

Break-down by clinical diagnosis confirmed that participants with AD demonstrated poorer global cognitive performance compared with other diagnostic groups, with lower mean MoCA (19.79 ± 5.35) and MMSE scores (22.29 ± 4.92), whereas the SCI group showed the highest cognitive scores across both measures (MoCA: 26.73 ± 3.77; MMSE: 27.45 ± 1.69; Supplementary Table 2).

**Figure 1:**
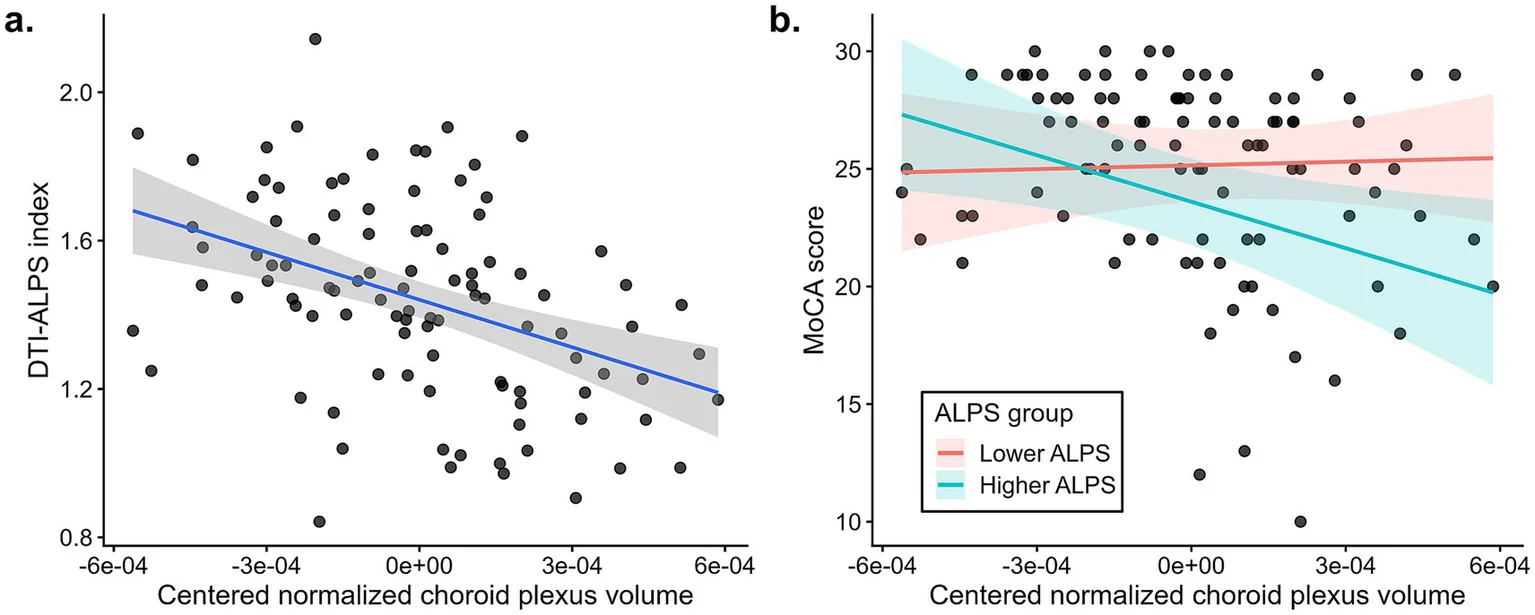
Associations between choroid plexus volume, DTI-ALPS, and cognitive performance. **(a)** Relationship between choroid plexus (CP) volume (normalized to the estimated total intracranial volume and zero-centered) and the diffusion along the perivascular space (DTI-ALPS) index. Larger CP volume was associated with lower DTI-ALPS values, indicating reduced diffusion along perivascular spaces. The solid line indicates the fitted linear regression and shaded band the 95% confidence interval. **(b)** Interaction between CP volume and DTI-ALPS in association with global cognitive performance. Predicted cognitive (Montreal Cognitive Assessment, MoCA) scores are shown across the range of CP volumes for lower versus higher DTI-ALPS values, illustrating the significant CP × DTI-ALPS interaction whereby the negative association between CP volume and cognition is stronger at higher ALPS values. Lines represent model-predicted values from the multivariable regression adjusted for age, sex, education, APOE ε4 status, WMH burden and plasma pTau217 levels. Shaded bands indicate 95% confidence intervals. Higher and lower ALPS groups were defined as mean ±1 SD.

To investigate whether structural alterations in the CP are associated with glymphatic function, we first examined the relationship between CP volume and the DTI-ALPS index using multivariable linear regression. After adjustment for age, sex, education, APOE ε4 status, and plasma pTau217 levels, larger CP volume was significantly associated with lower ALPS index (*β* = −282.19, *p* = 0.015; Figure 1a). This indicates that individuals with enlarged CP volumes exhibit reduced DTI-ALPS, which may suggest impaired glymphatic diffusion. Full regression outputs are provided in Supplementary Table 3, and model fit statistics in Supplementary Table 4.

### 3.2 Interaction between choroid plexus enlargement and DTI-ALPS is associated with cognitive performance

We next examined whether CP volume and DTI-ALPS were independently associated with global cognitive performance. In multivariable models adjusting for demographic variables and APOE ε4 status, neither CP volume nor ALPS index independently associated with MoCA scores. In contrast, plasma pTau217 levels were strongly associated with worse cognitive performance (*β* = −2.51, *p* < 0.001), indicating that higher AD pathological burden was associated with lower MoCA scores. The model explained approximately 28% of the variance in MoCA scores (*R*^2^ = 0.28, *p* < 0.001). These findings suggest that although AD pathology is strongly related to cognitive impairment, the independent contributions of CP structure and DTI-ALPS may be modest when considered separately. This raised the possibility that CP structure and DTI-ALPS may be associated with cognition through a joint rather than independent mechanism, so we therefore tested whether the two imaging markers interact to correlate with cognitive performance.

Including a CP × ALPS interaction term revealed a significant interaction effect (*β* = −13299.09, *p* = 0.036; Figure 1b). This indicates that the relationship between CP enlargement and cognitive performance depends on the level of DTI-ALPS. Specifically, the association between CP volume and MoCA scores became more negative at higher ALPS values. In individuals with relatively higher DTI-ALPS, larger CP volumes were associated with poorer cognitive performance, whereas the relationship between CP volume and cognition was weak or absent when ALPS values were lower. Incorporating the interaction term improved model performance (*R*^2^ = 0.31, *p* < 0.001), indicating that the combined effect of CP structure and DTI-ALPS explains additional variance in cognitive outcomes beyond models including main effects alone.

### 3.3 Sensitivity analyses

Because ventricular enlargement and increased CSF volume may reflect brain atrophy and could potentially confound relationships between CP volume and cognition, we conducted a sensitivity analysis including normalized CSF volume. In this model, the interaction between CP volume and ALPS index remained statistically significant (*β* = −13066.30, *p* = 0.038). Lower ALPS index was also independently associated with poorer MoCA scores (*β* = −4.15, *p* = 0.028), whereas CSF volume itself was not significantly associated with cognition. This model explained approximately 33% of the variance in MoCA scores (*R*^2^ = 0.33, *p* < 0.001), indicating that the CP-ALPS interaction is not explained by global CSF volume differences, and showing that the interaction findings remained significant after accounting for CSF (Δ*R*^2^ = 0.02; the difference between sensitivity and main interaction models). Sensitivity analysis to account for the potential confounding effect of WMH burden also showed that the main interaction effect remained significant when including normalized WMH as a covariate (Δ*R*^2^ = 0.01, *β* = −13758.5, *p* = 0.032).

To determine whether these findings generalized across cognitive measures and were not just specific to MoCA, we repeated the analysis using MMSE scores as the outcome. In this model, larger CP volume was associated with lower MMSE scores (*β* = −3594.05, *p* = 0.021). The interaction between CP volume and ALPS index was also significant (*β* = −13,388.70, *p* = 0.008), suggesting that the combined presence of CP enlargement and reduced DTI-ALPS was associated with poorer global cognitive performance. Higher plasma pTau217 levels were again strongly associated with worse cognition (*β* = −1.96, *p* < 0.001). The model explained approximately 32% of the variance in MMSE scores (*R*^2^ = 0.32, *p* < 0.001; interaction model difference Δ*R*^2^ = 0.01).

Finally, we examined whether plasma pTau217 modifies the relationship between CP volume, DTI-ALPS, and cognition using a three-way interaction model. The interaction between CP volume and ALPS index remained significant (*β* = −23286.13, *p* = 0.016), indicating that the CP-ALPS interaction persisted after accounting for plasma pTau217 levels. However, neither the CP × pTau217 nor ALPS×pTau217 interaction terms were significant, nor the three-way interaction between CP, ALPS, and pTau217. Sensitivity analyses stratified by plasma pTau217 risk strata suggested that the CP × ALPS interaction was marginally non-significant in participants with higher pTau217 levels, although these subgroup analyses should be interpreted cautiously given the limited sample size.

## 4 Discussion

**Figure 2:**
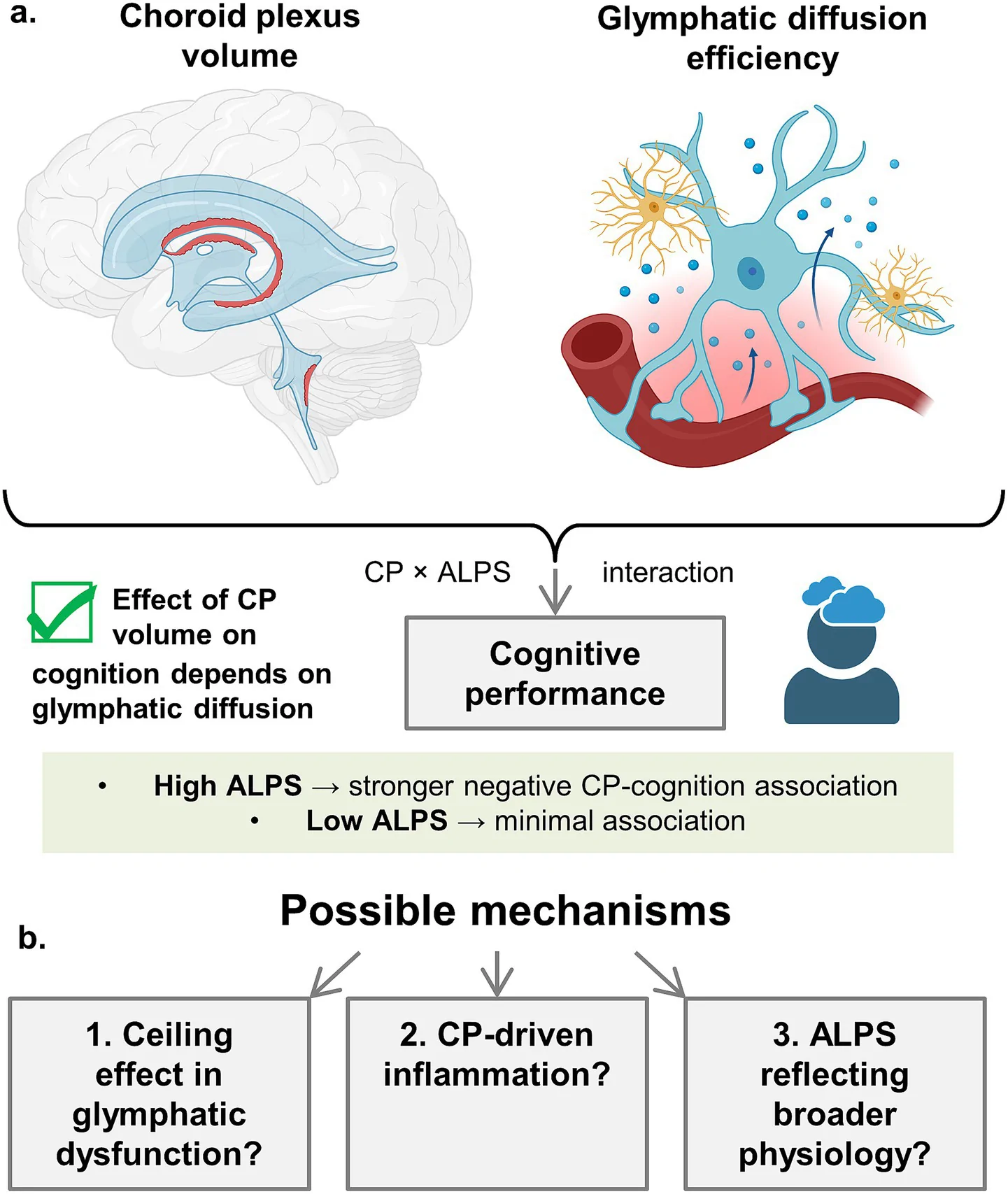
Conceptual model of the interaction between choroid plexus structure and glymphatic diffusion. **(a)** Choroid plexus enlargement was associated with reduced diffusion along the perivascular space (DTI-ALPS), suggesting altered brain fluid dynamics. However, cognitive performance was not independently associated with CP volume or ALPS index. Instead, a significant interaction was observed whereby the relationship between CP enlargement and cognition depended on glymphatic diffusion efficiency. **(b)** Three potential interpretations are proposed: (1) a ceiling effect whereby CP enlargement contributes little additional cognitive impact when glymphatic diffusion is already impaired; (2) inflammatory signaling associated with CP enlargement influencing cognition independently of glymphatic transport; and (3) the ALPS index reflecting broader vascular-perivascular physiology rather than glymphatic clearance alone. Created in BioRender.

Recent evidence suggests that dysfunction in CP and glymphatic systems may contribute to neurodegenerative processes and cognitive impairment, but their interaction is poorly understood. We therefore investigated the relationship between CP structure, DTI-ALPS as a proxy for glymphatic diffusion, and cognitive performance in a memory clinic population. In our sample, the interaction of CP volume and ALPS index significantly associated with cognitive outcomes despite neither being independently associated with cognitive performance after adjustment for demographic and molecular covariates. This indicates that that the relationship between CP structure and cognition depends on DTI-ALPS. This interaction remained significant after accounting for ventricular CSF volume and showed similar interaction effects using both MoCA and MMSE as cognitive outcomes, suggesting consistency across cognitive measures within this cohort. Together, our findings support the concept that structural alterations of the CP and glymphatic transport may interact to be associated with cognitive function (Figure 2). An apparently counterintuitive finding was that the negative association between CP volume and cognition was strongest at higher ALPS values. One possible explanation is that higher ALPS in this context does not reflect preserved glymphatic function, but rather compensatory or dysregulated perivascular fluid dynamics in response to CP-related pathology.

The observed association between CP enlargement and reduced ALPS index suggests a link between structural changes in the CP and impaired brain fluid dynamics. The CP is the primary site of CSF production and regulates exchange between the blood and CSF (Damkier et al., 2013). Enlargement of the CP has been reported across the AD continuum and has been linked to neuroinflammatory processes and altered CSF dynamics (Preziosa et al., 2025; Xu et al., 2024). Our findings extend this literature by demonstrating that CP structural alterations are also associated with diffusion-based measures related to glymphatic transport, suggesting that CP pathology may influence downstream CSF circulation and clearance processes.

A key observation of this study was that CP volume and ALPS index were not independently associated with cognition, but their interaction was significant. This indicates that CP structural changes may be associated with cognitive outcomes differently depending on the underlying state of brain fluid dynamics. In our data, individuals with higher ALPS had negative associations between CP volume and cognition, while individuals with lower ALPS had no association. Several studies using mediation analysis in neurodegenerative disease provide support for this framework (Lv et al., 2026; Ruan et al., 2026; Xu et al., 2024; Yu et al., 2025). One possible interpretation is the presence of a ceiling or floor effect in glymphatic diffusion: when ALPS values are already low, suggesting reduced glymphatic diffusion, cognitive impairment may already be driven by broader pathological processes, limiting the additional observable contribution of CP enlargement. In contrast, when glymphatic diffusion is relatively preserved, structural alterations of the CP may become more apparent as an independent contributor to cognitive dysfunction. In this context, CP enlargement may represent an early or parallel pathological process that interacts with fluid clearance pathways.

A second possible explanation relates to the role of the CP as an immunological interface between the peripheral circulation and the central nervous system, regulating the trafficking of immune cells (Dumas et al., 2024). Enlargement of the CP has been associated with inflammatory activation and blood-CSF barrier dysfunction in several neurological conditions (Althubaity et al., 2022; Reynolds and Putterman, 2025; Solár et al., 2020). From this perspective, CP enlargement may represent an inflammatory state that correlates with cognitive function through mechanisms that are partly independent of glymphatic transport itself.

A third consideration is that the ALPS index may reflect broader aspects of perivascular and vascular physiology rather than glymphatic clearance alone. DTI-ALPS likely captures multiple processes including perivascular diffusion, vascular pulsatility, and white matter microstructural properties (Taoka et al., 2024; Satpathi et al., 2025). Consequently, the interaction observed in this study may reflect complex relationships between CSF production, vascular dynamics, and perivascular fluid movement rather than a single clearance pathway.

Plasma pTau217 levels showed strong independent associations with cognitive performance, consistent with literature linking phosphorylated tau biomarkers to AD pathology and cognitive decline (Fernández Arias et al., 2025; Gonzalez-Ortiz et al., 2025; Malek-Ahmadi et al., 2026). Importantly, the interaction between CP volume and ALPS index remained significant after adjusting for plasma pTau217 levels, and neither CP × pTau217 nor ALPS×pTau217 interaction terms were significant. The CP-glymphatic interaction also persisted after accounting for ventricular CSF volume, suggesting that the findings are unlikely to be explained by global brain atrophy or ventricular enlargement. This indicates that the interaction between CP structure and glymphatic diffusion relates to cognitive performance independently of established AD pathology and structural brain changes.

Several limitations should be considered when interpreting these findings. First, the cross-sectional design limits causal inference. It remains unclear whether CP enlargement contributes to changes in glymphatic diffusion, whether impaired perivascular transport leads to CP alterations, or whether both processes reflect downstream consequences of neurodegenerative pathology. Furthermore, automated CP segmentation may be impacted by partial-volume effects and potential segmentation inaccuracies given the small size of the structure, despite our QC process. Longitudinal studies will be required to clarify the temporal relationships between these systems. Second, the precise biological interpretation of the ALPS index and its relation to glymphatic function remains an area of ongoing investigation. Third, the study sample was derived from a tertiary memory clinic population, which may limit generalisability to community populations or earlier stages of disease, but remains a strength of this study given the challenges faced when recruiting persons with cognitive impairment in a real-world memory clinic. Although we did test the sensitivity of our results to WMH burden, we could not account for all potential confounders such as sleep quality and other vascular risk factors.

## 5 Conclusion

This study provides novel evidence that structural changes in the CP and diffusion-based markers of perivascular transport interact in relation to cognitive function. By integrating structural MRI measures of the CP with diffusion-based indicators of brain fluid dynamics, our findings highlight the potential importance of interactions between CSF regulatory systems in neurodegenerative disease. Future studies combining multimodal imaging, molecular biomarkers, and longitudinal designs in a larger cohort across neurodegenerative diseases, including accounting for cerebrovascular pathology, may help clarify how disruption of the CP-glymphatic axis is associated with cognitive decline and whether these processes represent modifiable targets for therapeutic intervention.

## Data Availability

The raw data supporting the conclusions of this article will be made available by the authors, without undue reservation.
