# Correction: Choroid plexus enlargement is negatively associated with cognitive performance in a DTI-ALPS–dependent manner

**DOI:** 10.3389/fnagi.2026.1951916

**Published:** 2026-08-21

**Authors:** Nicole I. Y. Z. Tan, Thomas Welton, Yi Jayne Tan, Seyed Ehsan Saffari, Elenor Morgenroth, Sarah J. Y. Foo, Zi Rou Kwok, Yu Chin Lim, Hui Jin Chiew, Kok Pin Ng, Simon K. S. Ting, Sumeet Kumar, Nicole Keong, Adeline S. L. Ng

**Affiliations:** 1 Department of Neurology, National Neuroscience Institute, Singapore, Singapore; 2 Neuroscience Academic Clinical Programme, Duke NUS Medical School, Singapore, Singapore; 3 Lee Kong Chian School of Medicine, Nanyang Technological University, Singapore, Singapore; 4 Department of Neuroradiology, National Neuroscience Institute, Singapore, Singapore; 5 Department of Neurosurgery, National Neuroscience Institute, Singapore General Hospital, Singapore, Singapore

**Keywords:** Alzheimer's disease biomarkers, cerebrospinal fluid dynamics, choroid plexus, cognitive impairment, diffusion MRI, DTI-ALPS, glymphatic system, plasma pTau217

In the published article, the affiliation “Center for Biomedical Data Science, Duke-NUS Medical School, Singapore, Singapore” was erroneously included for author Seyed Ehsan Saffari. This affiliation has now been removed, and all subsequent affiliation numbers updated accordingly.

The original version of this article has been updated.

