# Supplementary material for: Choroid plexus enlargement is negatively associated with cognitive performance in a DTI-ALPS–dependent manner
Source: Front Aging Neurosci. 2026 Jul 27;18:1883244. doi: 10.3389/fnagi.2026.1883244 (PMC13454073; doi:10.3389/fnagi.2026.1883244)
Supplement: Supplementary file 1 [file Data_Sheet_1.DOCX]

Supplementary Material

## 1. Supplementary Figures

**Supplementary Figure 1.** ROIs were marked on an axial slice at the level of the body of the lateral ventricle. Each ROI comprised four voxels placed in a square, constituting 12.17mm^3^. Two ROIs were drawn per hemisphere in the projection and association area.

**Supplementary Figure 2.** Representative images showing choroid plexus segmentation (red) overlaid on three-dimensional T1-weighted magnetic resonance images in the axial (left), coronal (center), and sagittal (right) planes.

Patients from memory clinic (N = 100)

Patients with complete data (N = 96)

Excluded patients with missing data (N = 4) - APOE ε4 status only (N = 2) - Cognitive Scores (N = 1) - APOE ε4 status and cognitive scores (N =1)

**Supplementary Figure 3.** Participant flow diagram depicting participant inclusion and the availability of APOE ε4 status and cognitive data.

## 1.1 Supplementary Tables

|  | Overall | Low (<0.38) | Intermediate (0.38–0.66) | High (>0.66) |
| --- | --- | --- | --- | --- |
| N | 100 | 51 | 16 | 33 |
| Age, mean (SD) | 68.02 (7.91) | 67.12 (8.71) | 68.45 (8.91) | 69.19 (5.91) |
| Gender, number of males (%) | 50 (50.0) | 22 (43.1) | 13 (81.2) | 15 (45.5) |
| Education, mean (SD) | 13.27 (3.90) | 13.40 (4.40) | 13.25 (4.11) | 13.06 (2.97) |
| APOE genotype, number of ε4 carriers (%) | 29 (29.9) | 8 (16.3) | 4 (25.0) | 17 (53.1) |
| Choroid plexus volume mm3, mean (SD) | 1,715.18 (416.27) | 1,677.04 (413.78) | 1,750.46 (454.90) | 1,757.02 (408.44) |
| ALPS, mean (SD) | 1.44 (0.27) | 1.45 (0.25) | 1.40 (0.32) | 1.44 (0.27) |
| CSF volume mm3, mean (SD) | 40,282.89 (18,485.84) | 38,416.76 (19,646.84) | 43,410.31 (19,636.65) | 41,650.60 (16,154.71) |
| MoCA, mean (SD) | 24.67 (4.37) | 26.22 (2.96) | 25.19 (4.28) | 22.00 (5.06) |
| MMSE, mean (SD) | 26.32 (3.47) | 27.32 (2.53) | 27.19 (2.64) | 24.31 (4.25) |
| ptau217 plasma concentration pg/mL, mean (SD) | 0.68 (0.72) | 0.25 (0.07) | 0.46 (0.07) | 1.46 (0.81) |

**Supplementary Table 1 | Distribution of plasma pTau217 risk strata within the study cohort.** Participants were classified into low (<0.38 pg/mL), indeterminate (0.38–0.66 pg/mL), and high (>0.66 pg/mL) risk categories based on plasma pTau217 concentrations. Demographic and imaging characteristics across strata are presented descriptively.

|  | Overall | SCI | MCI | AD | VAD | FTD |
| --- | --- | --- | --- | --- | --- | --- |
| N | 100 | 11 | 70 | 15 | 3 | 1 |
| Age, mean (SD) | 68.02 (7.91) | 62.45 (7.58) | 69.05 (8.30) | 67.33 (5.25) | 68.92 (3.97) | 64.49 |
| Gender, number of males (%) | 50 (50.0) | 5 (45.5) | 35 (50.0) | 10 (66.7) | 0 (0.0) | 0 |
| Education, mean (SD) | 13.27 (3.90) | 15.00 (3.66) | 13.26 (3.98) | 12.47 (3.93) | 11.00 (1.73) | 13 |
| APOE genotype, number of ε4 carriers (%) | 29 (29.9) | 5 (45.5) | 17 (25.4) | 7 (46.7) | 0 (0.0) | 0 |
| Choroid plexus volume mm3, mean (SD) | 1,715.18 (416.27) | 1,512.87 (269.19) | 1,702.68 (431.68) | 1,867.56 (398.87) | 2,036.42 (319.34) | 1,566.28 |
| ALPS, mean (SD) | 1.44 (0.27) | 1.57 (0.16) | 1.43 (0.28) | 1.41 (0.24) | 1.28 (0.28) | 1.72 |
| CSF volume mm3, mean (SD) | 40,282.89 (18,485.84) | 30,001.29 (7,430.57) | 40,892.44 (20,419.05) | 44,462.85 (14,294.73) | 45,727.18 (4,212.43) | 31,679.82 |
| MoCA, mean (SD) | 24.67 (4.37) | 26.73 (3.77) | 25.42 (3.60) | 19.79 (5.35) | 23.67 (3.51) | 22 |
| MMSE, mean (SD) | 26.32 (3.47) | 27.45 (1.69) | 27.06 (2.72) | 22.29 (4.92) | 25.00 (3.46) | 23 |
| ptau217 plasma concentration pg/mL, mean (SD) | 0.68 (0.72) | 0.47 (0.27) | 0.60 (0.67) | 1.30 (0.95) | 0.58 (0.61) | 0.13 |

**Supplementary Table 2 | Clinical diagnostic composition of the study cohort.** Breakdown of participants by clinical diagnosis, including subjective cognitive impairment (SCI), mild cognitive impairment (MCI), Alzheimer’s disease (AD), vascular dementia (VaD), and frontotemporal dementia (FTD) diagnoses. Cognitive scores and demographic characteristics for each diagnostic group are summarized.

| model | n | r_squared | adj_r_squared | term | estimate | std_estimate | std.error | statistic | p.value | conf.low | conf.high |
| --- | --- | --- | --- | --- | --- | --- | --- | --- | --- | --- | --- |
| ALPS model | 97 | 0.269 | 0.2203 | (Intercept) | 1.7087 |  | 0.287 | 5.9528 | 5E-08 | 1.1384 | 2.2789 |
| ALPS model | 97 | 0.269 | 0.2203 | CP_c | -282.186 | -0.263 | 113.9223 | -2.477 | 0.01512 | -508.512 | -55.8595 |
| ALPS model | 97 | 0.269 | 0.2203 | Age | -0.0064 | -0.1846 | 0.0036 | -1.7768 | 0.07898 | -0.0137 | 0.0008 |
| ALPS model | 97 | 0.269 | 0.2203 | Gender1 | 0.1428 | 0.265 | 0.0521 | 2.7379 | 0.007451 | 0.0392 | 0.2464 |
| ALPS model | 97 | 0.269 | 0.2203 | Education | 0.0074 | 0.1074 | 0.0064 | 1.1519 | 0.2524 | -0.0054 | 0.0202 |
| ALPS model | 97 | 0.269 | 0.2203 | APOE41 | 0.0218 | 0.0371 | 0.0598 | 0.3651 | 0.7159 | -0.0969 | 0.1406 |
| ALPS model | 97 | 0.269 | 0.2203 | log_pTau217 | 0.0049 | 0.015 | 0.032 | 0.1516 | 0.8799 | -0.0588 | 0.0685 |
| MoCA main model | 96 | 0.278 | 0.2206 | (Intercept) | 19.8355 |  | 4.7824 | 4.1476 | 7.72E-05 | 10.3314 | 29.3396 |
| MoCA main model | 96 | 0.278 | 0.2206 | CP_c | -2425.97 | -0.1367 | 1949.17 | -1.2446 | 0.2166 | -6299.53 | 1447.598 |
| MoCA main model | 96 | 0.278 | 0.2206 | ALPS_c | -2.877 | -0.1779 | 1.7232 | -1.6696 | 0.09856 | -6.3016 | 0.5475 |
| MoCA main model | 96 | 0.278 | 0.2206 | Age | 0.0248 | 0.0438 | 0.0605 | 0.4093 | 0.6833 | -0.0955 | 0.145 |
| MoCA main model | 96 | 0.278 | 0.2206 | Gender1 | 0.0666 | 0.0076 | 0.891 | 0.0748 | 0.9406 | -1.704 | 1.8373 |
| MoCA main model | 96 | 0.278 | 0.2206 | Education | 0.0969 | 0.0865 | 0.1061 | 0.9131 | 0.3637 | -0.114 | 0.3077 |
| MoCA main model | 96 | 0.278 | 0.2206 | APOE41 | -0.2547 | -0.0267 | 0.9921 | -0.2567 | 0.798 | -2.2262 | 1.7168 |
| MoCA main model | 96 | 0.278 | 0.2206 | log_pTau217 | -2.5063 | -0.4687 | 0.5333 | -4.6992 | 9.58E-06 | -3.5662 | -1.4464 |
| MoCA interaction | 96 | 0.3137 | 0.2506 | (Intercept) | 18.5149 |  | 4.7302 | 3.9142 | 0.00018 | 9.1131 | 27.9166 |
| MoCA interaction | 96 | 0.3137 | 0.2506 | CP_c | -3033.1 | -0.1709 | 1932.385 | -1.5696 | 0.1201 | -6873.92 | 807.7272 |
| MoCA interaction | 96 | 0.3137 | 0.2506 | ALPS_c | -2.8869 | -0.1785 | 1.6897 | -1.7085 | 0.0911 | -6.2453 | 0.4716 |
| MoCA interaction | 96 | 0.3137 | 0.2506 | CP_ALPS | -13299.1 | -0.1938 | 6248.502 | -2.1284 | 0.03613 | -25718.7 | -879.515 |
| MoCA interaction | 96 | 0.3137 | 0.2506 | Age | 0.0373 | 0.066 | 0.0596 | 0.6255 | 0.5333 | -0.0812 | 0.1558 |
| MoCA interaction | 96 | 0.3137 | 0.2506 | Gender1 | -0.0169 | -0.0019 | 0.8745 | -0.0193 | 0.9846 | -1.7551 | 1.7213 |
| MoCA interaction | 96 | 0.3137 | 0.2506 | Education | 0.0983 | 0.0878 | 0.104 | 0.9445 | 0.3476 | -0.1085 | 0.305 |
| MoCA interaction | 96 | 0.3137 | 0.2506 | APOE41 | -0.2315 | -0.0243 | 0.9728 | -0.238 | 0.8125 | -2.165 | 1.702 |
| MoCA interaction | 96 | 0.3137 | 0.2506 | log_pTau217 | -2.6437 | -0.4944 | 0.5269 | -5.0172 | 2.75E-06 | -3.6911 | -1.5964 |
| MoCA + CSF sensitivity | 96 | 0.3334 | 0.2636 | (Intercept) | 20.379 |  | 4.8327 | 4.2169 | 6.1E-05 | 10.7718 | 29.9861 |
| MoCA + CSF sensitivity | 96 | 0.3334 | 0.2636 | CP_c | -1849.97 | -0.1042 | 2054.455 | -0.9005 | 0.3704 | -5934.09 | 2234.157 |
| MoCA + CSF sensitivity | 96 | 0.3334 | 0.2636 | ALPS_c | -4.1455 | -0.2563 | 1.8519 | -2.2385 | 0.02777 | -7.8271 | -0.464 |
| MoCA + CSF sensitivity | 96 | 0.3334 | 0.2636 | CP_ALPS | -13066.3 | -0.1904 | 6195.717 | -2.1089 | 0.03786 | -25383 | -749.622 |
| MoCA + CSF sensitivity | 96 | 0.3334 | 0.2636 | CSF_norm | -78.75 | -0.2026 | 49.4318 | -1.5931 | 0.1148 | -177.017 | 19.5172 |
| MoCA + CSF sensitivity | 96 | 0.3334 | 0.2636 | Age | 0.0499 | 0.0883 | 0.0596 | 0.837 | 0.4049 | -0.0686 | 0.1685 |
| MoCA + CSF sensitivity | 96 | 0.3334 | 0.2636 | Gender1 | 0.0515 | 0.0059 | 0.868 | 0.0593 | 0.9528 | -1.6739 | 1.7769 |
| MoCA + CSF sensitivity | 96 | 0.3334 | 0.2636 | Education | 0.0713 | 0.0637 | 0.1045 | 0.6824 | 0.4968 | -0.1364 | 0.279 |
| MoCA + CSF sensitivity | 96 | 0.3334 | 0.2636 | APOE41 | -0.2782 | -0.0292 | 0.9648 | -0.2884 | 0.7737 | -2.1961 | 1.6396 |
| MoCA + CSF sensitivity | 96 | 0.3334 | 0.2636 | log_pTau217 | -2.4724 | -0.4624 | 0.5333 | -4.6362 | 1.26E-05 | -3.5326 | -1.4123 |
| MMSE interaction | 96 | 0.3151 | 0.2521 | (Intercept) | 24.1758 |  | 3.7574 | 6.4343 | 6.51E-09 | 16.7076 | 31.6439 |
| MMSE interaction | 96 | 0.3151 | 0.2521 | CP_c | -3594.05 | -0.2547 | 1534.962 | -2.3415 | 0.02149 | -6644.95 | -543.144 |
| MMSE interaction | 96 | 0.3151 | 0.2521 | ALPS_c | -2.4827 | -0.1931 | 1.3422 | -1.8497 | 0.06775 | -5.1504 | 0.1851 |
| MMSE interaction | 96 | 0.3151 | 0.2521 | CP_ALPS | -13388.7 | -0.2454 | 4963.407 | -2.6975 | 0.00839 | -23254 | -3523.39 |
| MMSE interaction | 96 | 0.3151 | 0.2521 | Age | 0.0102 | 0.0226 | 0.0474 | 0.2143 | 0.8308 | -0.084 | 0.1043 |
| MMSE interaction | 96 | 0.3151 | 0.2521 | Gender1 | -0.4217 | -0.0605 | 0.6947 | -0.6071 | 0.5454 | -1.8025 | 0.959 |
| MMSE interaction | 96 | 0.3151 | 0.2521 | Education | -0.0234 | -0.0262 | 0.0826 | -0.2826 | 0.7782 | -0.1876 | 0.1409 |
| MMSE interaction | 96 | 0.3151 | 0.2521 | APOE41 | 0.1587 | 0.0209 | 0.7727 | 0.2054 | 0.8378 | -1.3772 | 1.6946 |
| MMSE interaction | 96 | 0.3151 | 0.2521 | log_pTau217 | -1.9609 | -0.4612 | 0.4186 | -4.6848 | 1.03E-05 | -2.7928 | -1.1289 |
| CP ALPS pTau217 interaction | 96 | 0.3304 | 0.2428 | (Intercept) | 18.0778 |  | 5.1163 | 3.5334 | 0.00067 | 7.9035 | 28.2521 |
| CP ALPS pTau217 interaction | 96 | 0.3304 | 0.2428 | CP_c | -4432.86 | -0.2498 | 2732.578 | -1.6222 | 0.1085 | -9866.89 | 1001.168 |
| CP ALPS pTau217 interaction | 96 | 0.3304 | 0.2428 | ALPS_c | -1.5923 | -0.0985 | 2.4612 | -0.647 | 0.5194 | -6.4866 | 3.3021 |
| CP ALPS pTau217 interaction | 96 | 0.3304 | 0.2428 | log_pTau217 | -2.8434 | -0.5318 | 0.5882 | -4.834 | 5.97E-06 | -4.0131 | -1.6737 |
| CP ALPS pTau217 interaction | 96 | 0.3304 | 0.2428 | Age | 0.0413 | 0.073 | 0.0632 | 0.6526 | 0.5158 | -0.0845 | 0.167 |
| CP ALPS pTau217 interaction | 96 | 0.3304 | 0.2428 | Gender1 | -0.2394 | -0.0273 | 0.893 | -0.2681 | 0.7893 | -2.0152 | 1.5363 |
| CP ALPS pTau217 interaction | 96 | 0.3304 | 0.2428 | Education | 0.1064 | 0.095 | 0.1069 | 0.9956 | 0.3223 | -0.1061 | 0.319 |
| CP ALPS pTau217 interaction | 96 | 0.3304 | 0.2428 | APOE41 | -0.391 | -0.041 | 1.0007 | -0.3907 | 0.697 | -2.381 | 1.599 |
| CP ALPS pTau217 interaction | 96 | 0.3304 | 0.2428 | CP_c:ALPS_c | -23286.1 | -0.3394 | 9501.346 | -2.4508 | 0.01633 | -42180.6 | -4391.66 |
| CP ALPS pTau217 interaction | 96 | 0.3304 | 0.2428 | CP_c:log_pTau217 | -1146.16 | -0.0739 | 2228.796 | -0.5143 | 0.6084 | -5578.37 | 3286.044 |
| CP ALPS pTau217 interaction | 96 | 0.3304 | 0.2428 | ALPS_c:log_pTau217 | 1.2144 | 0.0798 | 2.1184 | 0.5733 | 0.568 | -2.9982 | 5.427 |
| CP ALPS pTau217 interaction | 96 | 0.3304 | 0.2428 | CP_c:ALPS_c:log_pTau217 | -10595.5 | -0.1723 | 8528.868 | -1.2423 | 0.2176 | -27556.1 | 6365.073 |
| non AD sensitivity | 82 | 0.2392 | 0.1558 | (Intercept) | 21.0177 |  | 4.9447 | 4.2506 | 6.22E-05 | 11.1629 | 30.8724 |
| non AD sensitivity | 82 | 0.2392 | 0.1558 | CP_c | -1737.06 | -0.1214 | 1849.645 | -0.9391 | 0.3508 | -5423.4 | 1949.274 |
| non AD sensitivity | 82 | 0.2392 | 0.1558 | ALPS_c | -2.4895 | -0.1892 | 1.6519 | -1.5071 | 0.1361 | -5.7817 | 0.8027 |
| non AD sensitivity | 82 | 0.2392 | 0.1558 | CP_ALPS | -9567.45 | -0.1698 | 6068.728 | -1.5765 | 0.1192 | -21662.4 | 2527.511 |
| non AD sensitivity | 82 | 0.2392 | 0.1558 | Age | 0.0112 | 0.025 | 0.0592 | 0.1885 | 0.851 | -0.1069 | 0.1292 |
| non AD sensitivity | 82 | 0.2392 | 0.1558 | Gender1 | -0.5716 | -0.0789 | 0.8432 | -0.6779 | 0.5 | -2.2522 | 1.109 |
| non AD sensitivity | 82 | 0.2392 | 0.1558 | Education | 0.136 | 0.1472 | 0.1019 | 1.3338 | 0.1864 | -0.0672 | 0.3392 |
| non AD sensitivity | 82 | 0.2392 | 0.1558 | APOE41 | 0.2058 | 0.0252 | 0.9664 | 0.2129 | 0.832 | -1.7203 | 2.1319 |
| non AD sensitivity | 82 | 0.2392 | 0.1558 | log_pTau217 | -2.1089 | -0.4525 | 0.5407 | -3.9 | 0.000212 | -3.1865 | -1.0312 |
| AD sensitivity | 14 | 0.4279 | -0.4874 | (Intercept) | 14.5626 |  | 30.7592 | 0.4734 | 0.6559 | -64.5066 | 93.6317 |
| AD sensitivity | 14 | 0.4279 | -0.4874 | CP_c | -18077.1 | -0.6233 | 16264.22 | -1.1115 | 0.3169 | -59885.6 | 23731.44 |
| AD sensitivity | 14 | 0.4279 | -0.4874 | ALPS_c | 11.3275 | 0.5371 | 17.9034 | 0.6327 | 0.5547 | -34.6946 | 57.3496 |
| AD sensitivity | 14 | 0.4279 | -0.4874 | CP_ALPS | -98955 | -1.1809 | 83394.49 | -1.1866 | 0.2887 | -313327 | 115417.3 |
| AD sensitivity | 14 | 0.4279 | -0.4874 | Age | 0.1636 | 0.1662 | 0.4356 | 0.3756 | 0.7227 | -0.9561 | 1.2833 |
| AD sensitivity | 14 | 0.4279 | -0.4874 | Gender1 | -0.1353 | -0.0126 | 4.6853 | -0.0289 | 0.9781 | -12.1792 | 11.9085 |
| AD sensitivity | 14 | 0.4279 | -0.4874 | Education | -0.269 | -0.2017 | 0.5228 | -0.5146 | 0.6288 | -1.6129 | 1.0749 |
| AD sensitivity | 14 | 0.4279 | -0.4874 | APOE41 | -4.0882 | -0.3964 | 5.0443 | -0.8105 | 0.4545 | -17.055 | 8.8785 |
| AD sensitivity | 14 | 0.4279 | -0.4874 | log_pTau217 | 1.5401 | 0.1874 | 4.2229 | 0.3647 | 0.7302 | -9.3151 | 12.3954 |
| high ptau sensitivity | 31 | 0.2585 | 0.0329 | (Intercept) | 8.5534 |  | 13.2691 | 0.6446 | 0.5256 | -18.8958 | 36.0026 |
| high ptau sensitivity | 31 | 0.2585 | 0.0329 | CP_c | -6161.29 | -0.2921 | 4892.989 | -1.2592 | 0.2206 | -16283.2 | 3960.631 |
| high ptau sensitivity | 31 | 0.2585 | 0.0329 | ALPS_c | -2.2202 | -0.1203 | 5.5295 | -0.4015 | 0.6917 | -13.6588 | 9.2184 |
| high ptau sensitivity | 31 | 0.2585 | 0.0329 | CP_ALPS | -30984.7 | -0.4022 | 16904.71 | -1.8329 | 0.07979 | -65954.8 | 3985.324 |
| high ptau sensitivity | 31 | 0.2585 | 0.0329 | Age | 0.0938 | 0.1108 | 0.1991 | 0.4714 | 0.6418 | -0.318 | 0.5057 |
| high ptau sensitivity | 31 | 0.2585 | 0.0329 | Gender1 | 1.2326 | 0.1213 | 2.3 | 0.5359 | 0.5972 | -3.5253 | 5.9905 |
| high ptau sensitivity | 31 | 0.2585 | 0.0329 | Education | 0.373 | 0.2183 | 0.3422 | 1.0902 | 0.2869 | -0.3348 | 1.0809 |
| high ptau sensitivity | 31 | 0.2585 | 0.0329 | APOE41 | 1.0617 | 0.1045 | 2.0365 | 0.5213 | 0.6071 | -3.1511 | 5.2745 |
| low ptau sensitivity | 49 | 0.2192 | 0.0859 | (Intercept) | 28.285 |  | 4.7286 | 5.9817 | 4.59E-07 | 18.7354 | 37.8346 |
| low ptau sensitivity | 49 | 0.2192 | 0.0859 | CP_c | -2555.54 | -0.2043 | 2064.086 | -1.2381 | 0.2227 | -6724.05 | 1612.968 |
| low ptau sensitivity | 49 | 0.2192 | 0.0859 | ALPS_c | -5.0866 | -0.4418 | 1.8816 | -2.7033 | 0.009946 | -8.8866 | -1.2866 |
| low ptau sensitivity | 49 | 0.2192 | 0.0859 | CP_ALPS | -2071.36 | -0.0411 | 7584.556 | -0.2731 | 0.7861 | -17388.7 | 13245.96 |
| low ptau sensitivity | 49 | 0.2192 | 0.0859 | Age | -0.0543 | -0.156 | 0.0599 | -0.9055 | 0.3705 | -0.1753 | 0.0668 |
| low ptau sensitivity | 49 | 0.2192 | 0.0859 | Gender1 | -0.1846 | -0.0313 | 0.9408 | -0.1962 | 0.8454 | -2.0847 | 1.7154 |
| low ptau sensitivity | 49 | 0.2192 | 0.0859 | Education | 0.1311 | 0.1983 | 0.1029 | 1.2735 | 0.21 | -0.0768 | 0.3389 |
| low ptau sensitivity | 49 | 0.2192 | 0.0859 | APOE41 | -0.0362 | -0.0046 | 1.1969 | -0.0302 | 0.976 | -2.4534 | 2.3811 |
| WMH sensitivity | 96 | 0.3171 | 0.2456 | (Intercept) | 18.5235 |  | 4.746 | 3.903 | 0.000188 | 9.0888 | 27.9581 |
| WMH sensitivity | 96 | 0.3171 | 0.2456 | CP_c | -2985.58 | -0.1682 | 1940.198 | -1.5388 | 0.1275 | -6842.56 | 871.4095 |
| WMH sensitivity | 96 | 0.3171 | 0.2456 | ALPS_c | -2.5378 | -0.1569 | 1.7782 | -1.4272 | 0.1571 | -6.0727 | 0.9971 |
| WMH sensitivity | 96 | 0.3171 | 0.2456 | CP_ALPS | -13758.5 | -0.2005 | 6308.961 | -2.1808 | 0.03193 | -26300.3 | -1216.69 |
| WMH sensitivity | 96 | 0.3171 | 0.2456 | Age | 0.0332 | 0.0588 | 0.0602 | 0.552 | 0.5824 | -0.0864 | 0.1528 |
| WMH sensitivity | 96 | 0.3171 | 0.2456 | Gender1 | -0.1413 | -0.0161 | 0.898 | -0.1574 | 0.8753 | -1.9266 | 1.6439 |
| WMH sensitivity | 96 | 0.3171 | 0.2456 | Education | 0.1084 | 0.0968 | 0.1055 | 1.0268 | 0.3074 | -0.1014 | 0.3181 |
| WMH sensitivity | 96 | 0.3171 | 0.2456 | APOE41 | -0.1603 | -0.0168 | 0.9822 | -0.1632 | 0.8707 | -2.1128 | 1.7922 |
| WMH sensitivity | 96 | 0.3171 | 0.2456 | log_pTau217 | -2.6369 | -0.4932 | 0.5288 | -4.9868 | 3.16E-06 | -3.6881 | -1.5857 |
| WMH sensitivity | 96 | 0.3171 | 0.2456 | WMH_norm | 47.3634 | 0.0656 | 72.803 | 0.6506 | 0.5171 | -97.3643 | 192.091 |

**Supplementary Table 3 | Full regression model outputs for multivariable analyses.** Complete coefficient estimates, standard errors, test statistics, and p-values for all multivariable linear regression models examining relationships between choroid plexus volume, ALPS index, plasma pTau217, and cognitive outcomes. Models were adjusted for age, sex, education, and APOE ε4 carrier status.

| model | n | r_squared | adj_r_squared | sigma | f_statistic | df1 | df2 | model_p_value |
| --- | --- | --- | --- | --- | --- | --- | --- | --- |
| ALPS model | 97 | 0.269 | 0.2203 | 0.2391 | 5.5209 | 6 | 90 | 6.61E-05 |
| MoCA main model | 96 | 0.278 | 0.2206 | 3.8869 | 4.8403 | 7 | 88 | 0.00012 |
| MoCA interaction | 96 | 0.3137 | 0.2506 | 3.8112 | 4.9714 | 8 | 87 | 4.35E-05 |
| MoCA + CSF sensitivity | 96 | 0.3334 | 0.2636 | 3.778 | 4.7791 | 9 | 86 | 3.6E-05 |
| MMSE interaction | 96 | 0.3151 | 0.2521 | 3.0274 | 5.0037 | 8 | 87 | 4.03E-05 |
| CP ALPS pTau217 interaction | 96 | 0.3304 | 0.2428 | 3.8312 | 3.7687 | 11 | 84 | 0.000211 |
| non AD sensitivity | 82 | 0.2392 | 0.1558 | 3.3396 | 2.8693 | 8 | 73 | 0.007837 |
| AD sensitivity | 14 | 0.4279 | -0.4874 | 6.527 | 0.4675 | 8 | 5 | 0.8379 |
| high ptau sensitivity | 31 | 0.2585 | 0.0329 | 5.0561 | 1.1456 | 7 | 23 | 0.3704 |
| low ptau sensitivity | 49 | 0.2192 | 0.0859 | 2.8024 | 1.6445 | 7 | 41 | 0.1503 |
| WMH sensitivity | 96 | 0.3171 | 0.2456 | 3.8239 | 4.4367 | 9 | 86 | 8.66E-05 |

**Supplementary Table 4 | Model fit statistics for multivariable regression analyses.** Model diagnostics including R^2^, adjusted R^2^, F-statistics, and model p-values for all regression models reported in the manuscript.
